# Supplementary material for: A State Space Approach to Dynamic Modeling of Mouse-Tracking Data
Source: Front Psychol. 2019 Dec 17;10:2716. doi: 10.3389/fpsyg.2019.02716 (PMC6928115; doi:10.3389/fpsyg.2019.02716)
Supplement: Supplementary file 1 [file Data_Sheet_1.pdf]

Appendix A: Filtering and smoothing

The term $f(\mathbf{Z}|\mathbf{Y})$ in Eq. (11) is recursively computed given all the measurements up to the n-th step. Let:

$$\begin{aligned}\log f(z_{i,n}|\mathbf{y}_{ij,0:n},\theta) \propto \log f(y_{ijn}|z_{i,n},\theta)+ \\ + \log \int_{\mathbb{R}} f(z_{i,n}|z_{i,n-1},\theta) f(z_{i,n-1}|\mathbf{y}_{ij,0:n-1},\theta) dz_{i,n-1}\end{aligned} \tag{A.1}$$

be the filtering density at step n. The first term of the right-side of the equation is the observation equation whereas the second term represents the Chapman-Kolmogorov equation. Given the initialization at $n = 0$, the filter proceeds by solving the integral in the Chapman-Kolmogorov equation (prediction step) and compute the log posterior filtering density (update step). In our model, we solve Eq. (A.1) by means of a Gaussian approximation filter (Smith & Brown, 2003), which computes a gaussian approximation to the posterior density $f(z_{i,n}|\mathbf{y}_{ij,0:n},\theta)$ and determines its posterior mode $z_{i,n|n}$ and variance $\lambda^2_{i,n|n}$ recursively.

More technically, let:

$$\begin{aligned}\log f(z_{i,n}|\mathbf{y}_{ij,0:n},\theta) \propto \log f(y_{ijn}|z_{i,n},\theta)+ \\ + \log \int_{\mathbb{R}} f(z_{i,n}|z_{i,n-1},\theta) f(z_{i,n-1}|\mathbf{y}_{ij,0:n-1},\theta) dz_{i,n-1}\end{aligned} \tag{A.2}$$

be the filtering density at step n. Consider the following definitions:

$$y_{ijn}|z_{i,n} \sim mixVM(\mu_1,\mu_2,\kappa_1,\kappa_2,\pi_{ijn}) \tag{A.3}$$

$$z_{i,n}|z_{i,n-1} \sim \mathcal{N}(z_{i,n-1},\sigma^2_i) \tag{A.4}$$

$$z_{i,n-1}|\mathbf{y}_{ij,0:n-1} \sim \mathcal{N}(z_{i,n-1|n-1},\lambda^2_{i,n-1|n-1}) \tag{A.5}$$

where $z_{i,n-1|n-1}$ and $\lambda^2_{i,n-1|n-1}$ represent the mode and the variance of the gaussian approximation in the prediction step. Under definitions (A.4)-(A.5), integrating out for $z_{i,n-1}$ in Eq.(A.2) yields to:

$$z_{i,n}|\mathbf{y}_{ij,0:n-1},\theta \sim \mathcal{N}(z_{i,n-1|n-1},\lambda^2_{i,n-1|n-1}+\sigma^2_i) \tag{A.6}$$

For the sake of computational simplicity, we rewrite the measurement density in Eq. (2) as follows:

$$\begin{aligned}f(y_{ijn}|\pi_{ijn},\theta) = \left[\frac{\pi_{ijn}}{2\pi I_0(\kappa_1)}\exp\Big(\cos(y_{ijn}-\mu_1)^{\kappa_1}\Big)\right]^{u_{ijn}} \cdot \\ \cdot \left[\frac{1-\pi_{ijn}}{2\pi I_0(\kappa_2)}\exp\Big(\cos(y_{ijn}-\mu_2)^{\kappa_2}\Big)\right]^{1-u_{ijn}}\end{aligned} \tag{A.7}$$

where u_{ijn} is a (deterministic) indicator variable taking the value 1 when y_{ijn} is in the area of the screen associated to *C1*, and 0 when y_{ijn} is in the area associated to *C2* (see Fig. 1) (Banerjee et al., 2005). Next, using these results together with Eqs. (A.3)-(A.5) we obtain:

$$
\begin{aligned}
\log f(z_{i,n}|\mathbf{y}_{ij,0:n},\theta) \propto &\log f(y_{ijn}|z_{i,n},\theta) + \log f(z_{i,n}|\mathbf{y}_{ij,0:n-1},\theta)\\
&= u_{ijn}\big(\kappa_1\cos(y_{ijn}-\mu_1) + \log\pi_{ijn} - \log I_0(\kappa_1)\big)+\\
&+ (1-u_{ijn})\big(\kappa_2\cos(y_{ijn}-\mu_2) + \log(1-\pi_{ijn}) - \log I_0(\kappa_2)\big)+\\
&+ \frac{1}{2}\left(\log(\lambda^2_{i,n-1|n-1}+\sigma_i^2) - \frac{(z_{i,n}-z_{i,n|n-1})^2}{\lambda^2_{i,n-1|n-1}+\sigma_i^2}\right)
\end{aligned} \tag{A.8}
$$

Differentiate $\mathcal{F} \triangleq f(z_{i,n}|\mathbf{y}_{ij,0:n},\theta)$ around $z_{i,n|n}$ gives:

$$
\begin{aligned}
\frac{\partial \log\mathcal{F}}{\partial z_{i,n|n}} = \sum_{j=1}^{J}\Bigg(&\frac{2z_{i,n|n-1}-2z_{i,n|n}}{2\sigma_i^2+\lambda^2_{i,n-1|n-1}} + \frac{\exp\big(\beta_j - z_{i,n|n}\big)u_{ijn}}{\exp\big(\beta_j - z_{i,n|n}\big)+1} -\\
&- \frac{\exp\big(\beta_j - z_{i,n|n}\big)(u_{ijn}-1)}{\exp\big(2z_{i,n|n}-2\beta_j\big)-1}\Bigg)
\end{aligned} \tag{A.9}
$$

$$
\frac{\partial^2 \log\mathcal{F}}{\partial z^2_{i,n|n}} = \sum_{j=1}^{J}\left(-\frac{1}{4\cosh\big(\frac{1}{2}(\beta_j - z_{i,n|n})\big)^2} - \frac{1}{(\sigma_i^2+\lambda^2_{i,n-1|n-1})}\right) \tag{A.10}
$$

where $\cosh(x) \triangleq \big(1+\exp(-2x)\big)/\big(2\exp(-x)\big)$ is the hyperbolic cosine function.
Finally, the posterior moment $z_{i,n|n}$ is obtained by solving Eq. (A.9) whereas $\lambda^2_{i,n|n}$ is computed by the negative inverse of Eq. (A.10) (e.g., see: Tanner, 1991). As Eq. (A.9) is non-linear, it can be solved numerically (e.g., using the Broyden's method). The complete filtering algorithm is summarized in Table A.1.

Algorithm 1	Gaussian Approximation filter algorithm	
$\mathbf{n=0}$:	$z_{i,0}=0$	INITIALIZATION
	$\lambda^2_{i,0}=1$	
$\mathbf{n>0}$:	$z_{i,n\|n-1} = z_{i,n-1\|n-1}$	PREDICTION
	$\lambda^2_{i,n\|n-1} = \lambda^2_{i,n-1\|n-1}+\sigma_i^2$	
	$z_{i,n\|n} = \text{solve}\big(\partial\log f(z_{i,n}\|\mathbf{y}_{ij,0:n},\theta)/\partial z_{i,n\|n}\big)$	UPDATE
	$\lambda^2_{i,n\|n} = -\text{inv}\big(\partial^2\log f(z_{i,n}\|\mathbf{y}_{ij,0:n},\theta)/\partial z^2_{i,n\|n}\big)$	

Table A.1: Filtering algorithm on the interval $\{0,1,\dots,N\}$. The algorithm takes as input the parameters θ and the data $\{\mathbf{y}_{ij,0:N},\,\mathbf{u}_{ij,0:N}\}$ whereas returns as output the filtered states $\mathbf{z}_{i,0:N}$. Note that the notation $n|n-1$ indicates the prediction of the current n-th state given the previous $n-1$ whereas $n|n$ denotes the update of the predicted state.

Finally, to ensure that the unobserved sequence $\mathbf{z}_{i,0:N}$ is an approximate realization from $f(\mathbf{z}_{i,0:N}|\mathbf{y}_{ij,0:N})$ we need to refine the filtering results conditional on the whole observed data $\mathbf{y}_{ij,0:N}$. This task is

achieved by means of a standard fixed-interval smoothing algorithm (Ansley & Kohn, 1982; Mendel, 1995), which uses the posterior filtering moments $z_{i,n|n}$ and $\lambda^2_{i,n|n}$ as input. The smoothing algorithm is described in Table A.2.

Algorithm 2	Fixed-interval smoothing algorithm	
$\mathbf{n} = \mathbf{N}$:	$z_{i,N\|N} \sim \mathcal{N}(z_{i,N\|n}, \lambda^2_{i,N\|n})$	INITIALIZATION
	$\lambda^2_{i,N\|N} = 1$	
$\mathbf{n} < \mathbf{N}$:	$z_{i,n\|N} = z_{i,n\|n} + \left(\frac{\lambda^2_{i,n\|n}}{\lambda^2_{i,n+1\|n}}\right)(z_{i,n+1\|N} - z_{i,n+1\|n})$	BACKWARD UPDATE
	$\lambda^2_{i,n\|N} = \lambda^2_{i,n\|n} + \left(\frac{\lambda^2_{i,n\|n}}{\lambda^2_{i,n+1\|n}}\right)^2 (\lambda^2_{i,n+1\|N} - \lambda^2_{i,n+1\|n})$	

Table A.2: Backward smoothing algorithm over the interval $\{N, N-1, \ldots, 0\}$. The algorithm takes as input the filtering solutions whereas returns as output the smoothed states $\mathbf{z}_{i,0:N}$ conditional on the whole set of data $\mathbf{y}_{ij,0:N}$. Note that the the notation $.|n$ refers to the filtering solutions whereas $.|N$ indicates the smoothing results.

Appendix B: Posteriors computation and estimation

In what follows, we describe the steps for computing the term $f(\boldsymbol{\Theta}|\mathbf{Y})$. First, we note that the array $\boldsymbol{\Theta}$ consists of two blocks of parameters associated to the observation equation and the stimuli equation of the model (3)-(4), namely J scalars $\{\beta_1, \ldots, \beta_J\}$ paired with the set of stimuli/trials and two parameters $\{\kappa_1, \kappa_2\}$ for the vonMises concentrations. To simplify the computations in the Metropolis-Hastings algorithm, the terms $\{\kappa_1, \kappa_2\}$ can be distinctively determined prior running the MH algorithm (Marin et al., 2005). Moreover, since we are not interested in the posterior distributions of these parameters, as long as they are not involved in the state-space dynamics, we compute them using the following Maximum-Likelihood estimators (Banerjee et al., 2005):

$$\hat{\kappa}_1 = I^{-1}\left(\frac{\sum_{i=1}^{I}\sum_{j=1}^{J}\sum_{n=0}^{N} u_{ijn}\cos(y_{ijn}-\mu_1)}{\sum_{i=1}^{I}\sum_{j=1}^{J}\sum_{n=0}^{N} u_{ijn}}\right) \tag{A.11}$$

$$\hat{\kappa}_2 = I^{-1}\left(\frac{\sum_{i=1}^{I}\sum_{j=1}^{J}\sum_{n=0}^{N} (1-u_{ijn})\cos(y_{ijn}-\mu_2)}{\sum_{i=1}^{I}\sum_{j=1}^{J}\sum_{n=0}^{N} (1-u_{ijn})}\right) \tag{A.12}$$

where μ_1 and μ_2 are the location parameters fixed by the experimenter, u_{ijn} is defined as in Eq. (A.7), whereas I^{-1} is the inverse of the modified Bessel function which is evaluated numerically (Abramowitz et al., 1972). Given the above results and the constrains $\boldsymbol{\sigma}_{I\times1} = \mathbf{1}_I$, the array of parameters simply reduces to $\boldsymbol{\theta}_{J\times1}$. This simplifies the inner-working of the MH algorithm as it now works on a smaller and more compact parameter space. To proceed further, the decomposition (10) involves the following definition for the MH proposal density (Andrieu et al., 2010):

$$q(\{\boldsymbol{\theta}^{(t)}, \mathbf{Z}^{(t)}\}|\{\boldsymbol{\theta}^{(t-1)}, \mathbf{Z}^{(t-1)}\}) \;=\; q(\boldsymbol{\theta}^{(t)}|\boldsymbol{\theta}^{(t-1)}) f(\mathbf{Z}^{(t)}|\mathbf{Y}) \tag{A.13}$$

where $f(\mathbf{Z}^{(t)}|\mathbf{Y})$ is evaluated through filtering/smoothing. This is appealing since the posterior density $f(\mathbf{Z}, \boldsymbol{\Theta}|\mathbf{Y})$ from which it might be difficult to sample from, reduces now to $f(\boldsymbol{\Theta}|\mathbf{Y})$ that is conveniently defined on a smaller parameters space (Andrieu et al., 2009). In our case, we can set:

$$q(\boldsymbol{\theta}^{(t)}|\boldsymbol{\theta}^{(t-1)}) \;=\; \mathcal{N}(\boldsymbol{\theta}^{(t-1)}, \boldsymbol{\Sigma}^{(t)}) \tag{A.14}$$

with $\boldsymbol{\Sigma}^{(t)}$ being a suitable $J \times J$ covariance matrix. Consequently, the MH acceptance ratio is as follows:

$$\alpha^{(t)} \;=\; \frac{f(\mathbf{Y}|\boldsymbol{\theta}^{(t)})\; q(\boldsymbol{\theta}^{(t-1)}|\boldsymbol{\theta}^{(t)})\; f(\boldsymbol{\theta}^{(t)})}{f(\mathbf{Y}|\boldsymbol{\theta}^{(t-1)})\; q(\boldsymbol{\theta}^{(t)}|\boldsymbol{\theta}^{(t-1)})\; f(\boldsymbol{\theta}^{(t-1)})} \tag{A.15}$$

where $f(\mathbf{Y}|\boldsymbol{\theta})$ is the density for the marginal likelihood computation, $f(\boldsymbol{\theta})$ indicates the prior density over the parameters, whereas $q(\boldsymbol{\theta}^{(t)}|\boldsymbol{\theta}^{(t-1)})$ is the MH proposal density.[5] Note that under (A.14), the terms $q(\boldsymbol{\theta}^{(t)}|\boldsymbol{\theta}^{(t-1)})$ and $q(\boldsymbol{\theta}^{(t-1)}|\boldsymbol{\theta}^{(t)})$ in Eq. (A.15) vanish as they refer to the same probability value. This yields to:

$$\alpha^{(t)} \;=\; \frac{f(\mathbf{Y}|\boldsymbol{\theta}^{(t)})\; f(\boldsymbol{\theta}^{(t)})}{f(\mathbf{Y}|\boldsymbol{\theta}^{(t-1)})\; f(\boldsymbol{\theta}^{(t-1)})} \tag{A.16}$$

where the MH ratio is now expressed as a function of the marginal likelihood and the priors. The term $f(\mathbf{Y}|\boldsymbol{\theta})$ can be easily computed as a byproduct of the filtering calculations (see Appendix C). Finally, the choice of a well-suited covariance matrix $\boldsymbol{\Sigma}^{(t)}$ for the proposal distribution is crucial in order to achieve chains' convergences. In our context, we used the Haario's adaptive solution where the proposal covariance is iteratively adapted during the chains using the current proposal covariance up to the adaptation step (Haario et al., 2001). The complete MH algorithm is summarized in Table A.3.

Appendix C: Marginal Likelihood computation

The marginal likelihood $f(\mathbf{Y}|\boldsymbol{\theta})$ is computed as follows. Let the observed-data log likelihood be:

$$\begin{aligned}
\log \mathcal{L}(\theta|\mathbf{Y}) &= \sum_{i=1}^{I}\sum_{j=1}^{J} \log f(\mathbf{y}_{ij,0:n}|\theta) \\
&= \sum_{i=1}^{I}\sum_{j=1}^{J}\left(\sum_{n=0}^{N} \log f(y_{ijn}|\mathbf{y}_{ij,0:n-1}, \theta)\right) \\
&= \sum_{i=1}^{I}\sum_{j=1}^{J}\left(\sum_{n=0}^{N} \log \int_{\mathbb{R}} f(y_{ijn}|z_{i,n}, \theta) f(z_{i,n}|\mathbf{y}_{ij,0:n-1}, \theta)\; dz_{i,n}\right)
\end{aligned} \tag{A.17}$$

[5]Another possibility to define the proposal density for the Metropolis Hasting algorithm is that of using an efficient fully adaptive mixture proposal density for multi-modal and multi-dimensional target distributions as described by Luengo & Martino (2013).

Algorithm 3	Metropolis-Hasting algorithm	
$\mathbf{t = 0}$:	Set $\boldsymbol{\theta}^{(t)} = \boldsymbol{\theta}^{(0)}$, $\boldsymbol{\Sigma}^{(t)} = \boldsymbol{\Sigma}^{(0)}$	INITIALIZATION
	Run Algorithms 1-2 to get $\mathbf{Z}^{(0)} \sim f\big(\mathbf{Z}\vert\mathbf{Y}, \boldsymbol{\theta}^{(\mathbf{0})}\big)$	
	Compute $f(\mathbf{Y}\vert\boldsymbol{\theta}^{(\mathbf{0})})$	
$\mathbf{t > 0}$:	$\boldsymbol{\theta}^{\star} \sim \mathcal{N}(\boldsymbol{\theta}^{(t-1)}, \boldsymbol{\Sigma}^{(t)})$	M-H LOOP
	Run Algorithms 1-2 to get $\mathbf{Z}^{\star} \sim f\big(\mathbf{Z}\vert\mathbf{Y}, \boldsymbol{\theta}^{\star}\big)$	
	Compute $f(\mathbf{Y}\vert\boldsymbol{\theta}^{\star})$	
	Compute $\alpha^{(t)}$ from Eq. (A.16)	
	Get $r \sim \mathcal{U}(0,1)$	ACCEPT/REJECT
	Set $\boldsymbol{\theta}^{(t)} = \boldsymbol{\theta}^{\star}$, $\mathbf{Z}^{(t)} = \mathbf{Z}^{\star}$ if $\alpha^{(t)} \leq r$	
	Set $\boldsymbol{\theta}^{(t)} = \boldsymbol{\theta}^{(t-1)}$, $\mathbf{Z}^{(t)} = \mathbf{Z}^{(t-1)}$ if $\alpha^{(t)} > r$	
	Run $\boldsymbol{\Sigma}^{(t+1)} \leftarrow \text{adapt}\big(\boldsymbol{\Sigma}^{(t)}\big)$ see Haario et al. (2001)	ADAPTING PHASE

Table A.3: Marginal Metropolis-Hastings to estimate $\boldsymbol{\beta}_{J\times 1}$. Note that $\mathcal{U}(0,1)$ indicates the Uniform distribution over the interval $[0,1]$ whereas the adaptive phase can be performed at each iteration t of the chain or rather after a fixed interval $t+H$ (with $H > 1$).

According to the standard prediction error decomposition, this functional has been factorized as a function of both the measurement model density (2) and the one-step ahead predictive density (A.6) (De Jong, 1988). By substituting the above definitions with our model's densities, we get the following marginal likelihood:

$$\log \mathcal{L}(\theta|\mathbf{Y}) = \sum_{i=1}^{I}\sum_{j=1}^{J}\sum_{n=0}^{N} \log\Bigg(\int_{\mathbb{R}} \big(\pi_{ijn}\exp(\cos(y_{ijn}-\mu_1)^{\kappa_1}\big)^{u_{ijn}} \cdot \\ \cdot \big((1-\pi_{ijn})\exp(\cos(y_{ijn}-\mu_2)^{\kappa_2}\big)^{(1-u_{ijn})} \cdot \\ \cdot \exp\left(-\frac{(z_{i,n}-z_{i,n|n-1})^2}{2(\sigma_i^2+\lambda^2_{i,n-1|n-1})}\right)\, dz_{i,n}\Bigg) \tag{A.18}$$

where the von-Mises and Gaussian densities have been written by dropping the constant terms. Using the predictive moments $z_{i,n|n-1}$ and $\lambda^2_{i,n|n-1}$ from the filter algorithm (see Table A.1), a recursion on $\{0,1,\ldots,N\}$ can be written for the likelihood computation, which consists of solving N integrals over the support of the r.v. $Z_{i,n}$. Since no analytical solutions are available for the functional above, numerical integration methods can be used to solve the integrals (e.g., see Shampine, 2008). Another possibility to compute the marginal likelihood is that of using a conditional Monte Carlo approximation by taking the advantages of the conditional equations of the models, in the spirit of Cabras et al. (2015).
