# Supplementary material for: A State Space Approach to Dynamic Modeling of Mouse-Tracking Data
Source: Front Psychol. 2019 Dec 17;10:2716. doi: 10.3389/fpsyg.2019.02716 (PMC6928115; doi:10.3389/fpsyg.2019.02716)
Supplement: Supplementary file 2 [file Data_Sheet_2.pdf]

Supplementary Materials

A state space approach to dynamic modeling of mouse-tracking data

Antonio Calcagnì°,* Luigi Lombardi†, Marco D'Alessandro†, Francesca Freuli†
°University of Padova, †University of Trento

Contents

1 Evaluating the state-space model **2**
1.1 Computation time 2
1.2 Simulation study 2
1.2.1 Simulation design 3
1.2.2 Outcome measures 4
1.2.3 Results 4

2 Case study **5**
2.1 Convergences of the MCMC chains 5
2.2 Fit of the model 7

---

*Corresponding author: antonio.calcagni@unipd.it

1 Evaluating the state-space model

1.1 Computation time

In this section we evaluate the time required by the algorithm included in Table A.3 of the manuscript to completely fit the state-space model. The algorithm has been implemented in Matlab R2018a and it is freely available online at `https://github.com/antcalcagni/SSM_mousetracking`. We tested two instances of our model, namely the simple model with a categorical variable and the complete model including categorical and continuous variables together with their interaction. The simulation study was obtained by varying the type of model (*categorical*, *interaction*), the number of statistical units $I = (12, 25, 50)$, the number of trials $J = (12, 28)$ and the number of levels of the categorical variable $K = (2, 4)$. The time step was kept fixed $N = 61$. The study involved $I \times J \times K = 12$ scenario for two type of models (see Table 1). For each scenario, data and model structure were kept fixed, one single chain was used without parallelization, warming-up and sampling periods were equal to 2000 samples (warm-up = 1000 samples), and the adaptive parameter of the MH-loop was equal to $H = 25$. The elapsed time was measured in seconds using the standard built-in Matlab function. Computations were performed on Intel Core i7-7500U 4-core 2.70GHz, Ubuntu 18.04 64-bit, 8GB Ram. Figure 1 shows the results for both types of models. As expected, the elapsed time increased exponentially as a function of the complexity of the scenario. In both models, the most simple scenario (scenario no. 1) required less then one minute to complete the warming-up and about one minute to complete the sampling period. Instead, the medium scenario (scenario no. 6) required about one minute to complete the warming-up and less then three minutes to complete the sampling phase. Finally, the most complex scenario (scenario no. 12) required less than three minutes to finalize the warming-up and about seven minutes to complete the sampling period.

scenario	parameters
1	I=12,J=12,K=2
2	I=12,J=12,K=4
3	I=12,J=28,K=2
4	I=12,J=28,K=4
5	I=25,J=12,K=2
6	I=25,J=12,K=4
7	I=25,J=28,K=2
8	I=25,J=28,K=4
9	I=50,J=12,K=2
10	I=50,J=12,K=4
11	I=50,J=28,K=2
12	I=50,J=28,K=4

Table 1: Computation time: Simulated scenario for both categorical and interaction models

1.2 Simulation study

In this section, we report the results of a simulation study to evaluate the ability of the model to (i) *recover* the true parameters $\boldsymbol{\beta}$ and (ii) *resemble* the observed data $\mathbf{Y}$. We designed the simulation under the Bayesian framework of statistical modeling. In particular, four factors were varied in a complete factorial design with 16 scenarios: type of model = {categorical, interaction}, $I = \{12, 25\}$, $J = \{12, 28\}$, $K = \{2, 4\}$. The combinations type of model $\times\, I \times J \times K$ were evaluated $M = 5000$

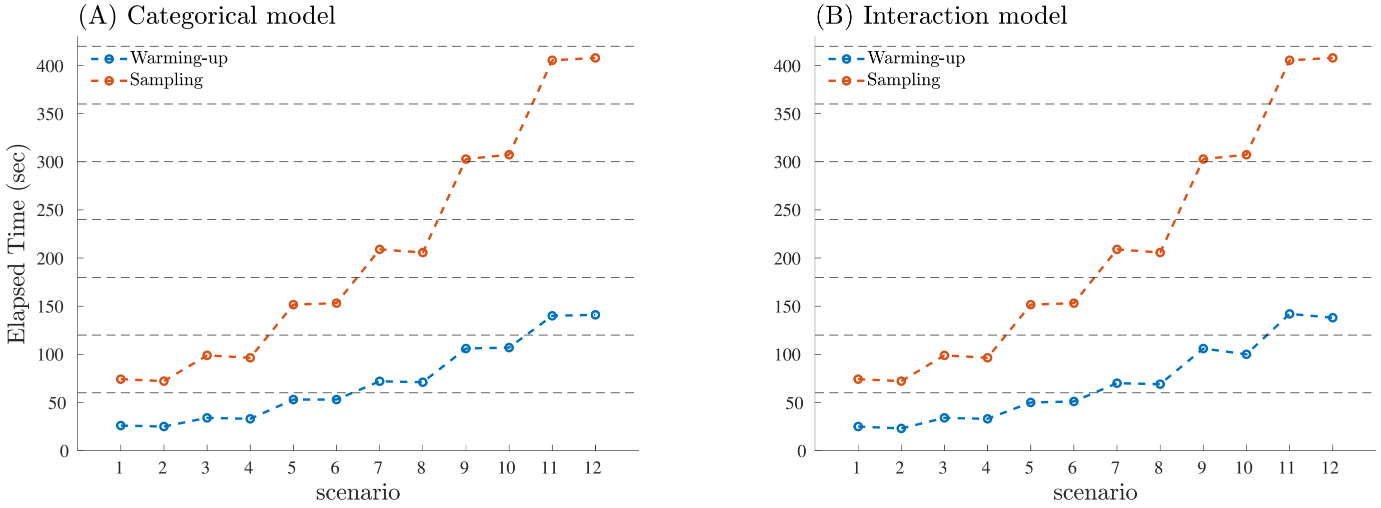

Figure 1: Computation time: Elapsed time (sec.) with regards to warming-up (blue line) and sampling (red line) for the simplest categorical (A) and interaction (B) models. Note that dotted gray lines indicate minutes.

times by getting a total of 40000 final samples. The levels of I, J, and K were chosen to represent the number of individuals, trials, and variables commonly encountered in experimental psychology. The simulation was performed on a (remote) HPC machine based on 16 cpu Intel Xeon CPU E5-2630L v3 1.80GHz, 16x4GB Ram.

1.2.1 Simulation design

The simulation workflow proceeded as follows:

1. The hyperparameters $\boldsymbol{\mu}_\beta$ and $\boldsymbol{\Sigma}_\beta$ of the prior $f(\boldsymbol{\beta}) = \mathcal{N}(\boldsymbol{\mu}_\beta, \boldsymbol{\Sigma}_\beta)$ were kept fixed, $\mu_1 = 2.75$, $\mu_2 = 0.75$, $\kappa_1 = \kappa_2 = 100$, $X \sim \mathcal{U}_d(-3, 3)$, $\mathbf{D} = \mathbf{I}_{K\times 1} \otimes \mathbf{1}_{\frac{J}{K}\times 1}$ (with $\otimes$ being the Kronecker product)

2. For each scenario $s = 1, \ldots, 16$, the parameters were sampled from the priors $\boldsymbol{\beta}_{1,\ldots,M} \sim f(\boldsymbol{\beta})$ and a series of M datasets $\mathbf{Y}_1^s, \ldots, \mathbf{Y}_M^s$ were generated according to the model equations (note that the generic dataset is of the form $\mathbf{Y}^s_{I\times J\times N}$)

3. For each of the datasets $\mathbf{Y}_1^s, \ldots, \mathbf{Y}_M^s$, the model was fitted by obtaining the array of latent states and parameter posteriors:

$$\mathcal{R} = \{(\mathbf{Z}_1^s, \ldots, \mathbf{Z}_M^s), (f(\boldsymbol{\beta}|\mathbf{Y})_1^s, \ldots, f(\boldsymbol{\beta}|\mathbf{Y})_M^s)\}$$

   where the generic array $\mathbf{Z}^s$ is of the form $\mathbf{Z}^s_{I\times N}$. The model was run using 10000 (samples) x 8 (chains), with warming-up of 2500 samples. Matlab Parallel computing was used to run the chains.

1.2.2 Outcome measures

The *recovery problem* was evaluated for $s = 1, \ldots, 16$ by means of the overlapping measure between symmetric distributions:

$$\Lambda\Big(f(\boldsymbol{\beta})^s, f(\boldsymbol{\beta}|\mathbf{Y})^s_{1,\ldots,M}\Big) = \frac{1}{M}\sum_{m=1}^{M}\int_{\mathbb{R}^n} \min\Big(f(\boldsymbol{\beta})^s, f(\boldsymbol{\beta}|\mathbf{Y})^s_m\Big)$$

which measures the amount of agreement between two distributions, with $\Lambda = 0$ indicating no overlapping. In our context, as $f(\boldsymbol{\beta})$ and $f(\boldsymbol{\beta}|\mathbf{Y})$ are symmetric with a unique mode, we interpret Λ as density similarity where $\Lambda = 1$ means that the two distributions are the same[1].

The *resembling problem* was instead assessed by means of the $\mathbf{Y}|\tilde{\mathbf{Y}}$-discrepancy measure:

$$\overline{\text{AoR}}^s = \frac{1}{M}\sum_{m=1}^{M}\text{AoR}\Big(\mathbf{Y}^s_m, \tilde{\mathbf{Y}}^s_m\Big)$$

where:

$$\text{AoR}\Big(\mathbf{Y}, \tilde{\mathbf{Y}}\Big) = 100 \cdot \frac{\min\big(||\mathbf{y}^\dagger||, ||\text{vec}(\mathbf{Y})||\big)}{\max\big(||\mathbf{y}^\dagger||, ||\text{vec}(\mathbf{Y})||\big)} \quad \text{with} \quad \mathbf{y}^\dagger = \frac{\text{vec}(\tilde{\mathbf{Y}})^T\text{vec}(\mathbf{Y})}{||\text{vec}(\mathbf{Y})||} \cdot \frac{\text{vec}(\mathbf{Y})}{||\text{vec}(\mathbf{Y})||}$$

which measures the *amount of reconstruction*[2] of the observed array $\mathbf{Y}$ via the reproduced array $\tilde{\mathbf{Y}}$. The measure AoR takes values in [0,100] with 0% indicating that no reconstruction occurred at all.

1.2.3 Results

The results of the simulation study are described in Tables 2-3. Overall, both the models got acceptable fit with regard to recover the true model structure (Λ index) and the adequacy in reproducing the input data (AoR index). As expected, the goodness-of-fit increased as a function of the number of trials J and the number of individuals I.

		AoR	Λ_{γ_1}	Λ_{γ_2}	Λ_{γ_3}	Λ_{γ_4}
$I = 12$	$J = 12, K = 2$	0.688	0.816	0.790		
	$J = 12, K = 4$	0.746	0.825	0.862	0.853	0.794
	$J = 28, K = 2$	0.732	0.828	0.848		
	$J = 28, K = 4$	0.799	0.874	0.807	0.830	0.831
$I = 25$	$J = 12, K = 2$	0.780	0.848	0.796		
	$J = 12, K = 4$	0.808	0.749	0.841	0.805	0.895
	$J = 28, K = 2$	0.753	0.809	0.844		
	$J = 28, K = 4$	0.811	0.828	0.866	0.833	0.857

Table 2: Simulation study (Categorical model): AoR and Λ indices for each level of the simulation design.

[1]Schmid, F., & Schmidt, A. (2006). Nonparametric estimation of the coefficient of overlapping—theory and empirical application. *Computational statistics & data analysis*, 50(6), 1583-1596.

[2]Sadtler, P. T., Quick, K. M., et al. (2014). Neural constraints on learning. *Nature*, 512(7515), 423-426.

		AoR	Λ_{γ_1}	Λ_{γ_2}	Λ_{γ_3}	Λ_{γ_4}	Λ_{η}	Λ_{δ_1}	Λ_{δ_2}	Λ_{δ_3}
$I = 12$	$J = 12, K = 2$	0.695	0.809	0.843			0.637	0.841		
	$J = 12, K = 4$	0.720	0.909	0.880	0.943	0.754	0.685	0.794	0.872	0.827
	$J = 28, K = 2$	0.807	0.813	0.831			0.684	0.835		
	$J = 28, K = 4$	0.710	0.955	0.946	0.863	0.902	0.674	0.933	0.888	0.900
$I = 25$	$J = 12, K = 2$	0.751	0.816	0.843			0.737	0.789		
	$J = 12, K = 4$	0.861	0.919	0.893	0.885	0.910	0.675	0.838	0.897	0.835
	$J = 28, K = 2$	0.794	0.862	0.842			0.701	0.762		
	$J = 28, K = 4$	0.873	0.875	0.848	0.981	0.806	0.726	0.887	0.870	0.869

Table 3: Simulation study (Interaction model): AoR and Λ indices for each level of the simulation design.

2 Case study

2.1 Convergences of the MCMC chains

In this section we illustrate the convergence diagnostics for the three case studies described in Section 4 of the manuscript.

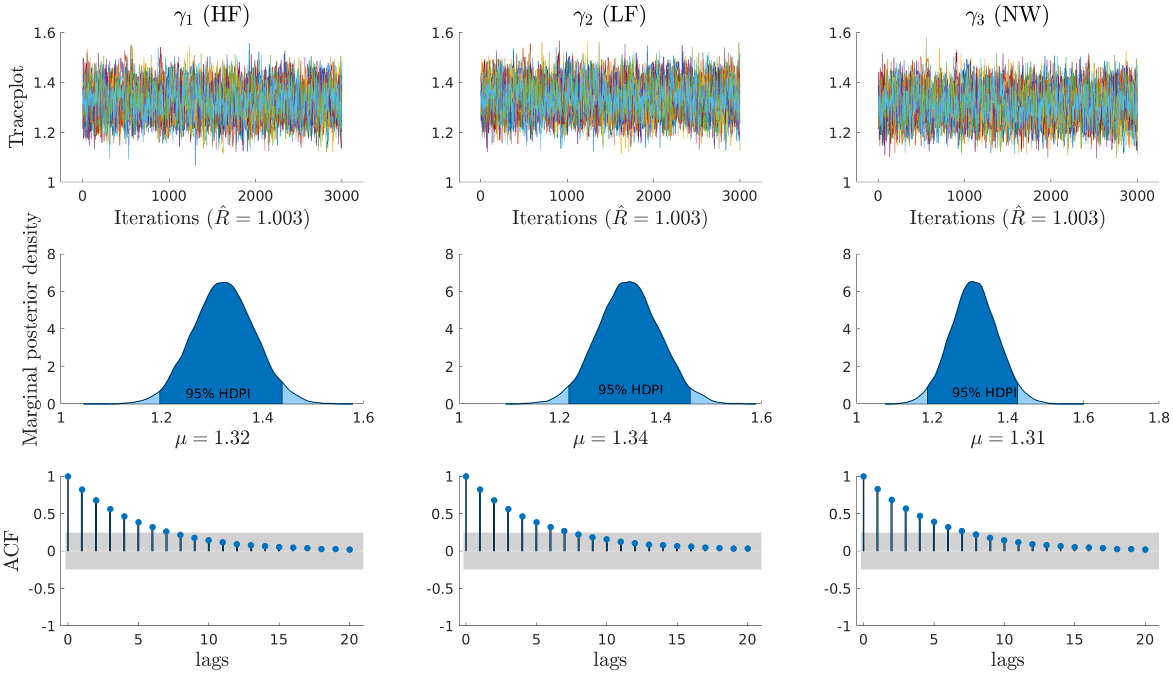

Figure 2: Case study (Model 2): MCMC diagnostics. Left panel: Traceplot of the chains in different colors and Gelman-Rubin $\hat{R}$ index. Middle panel: Marginal posterior density with posterior means μ and 95% HDPI. Right panel: Autocorrelation function (ACF) for the collapsed chains.

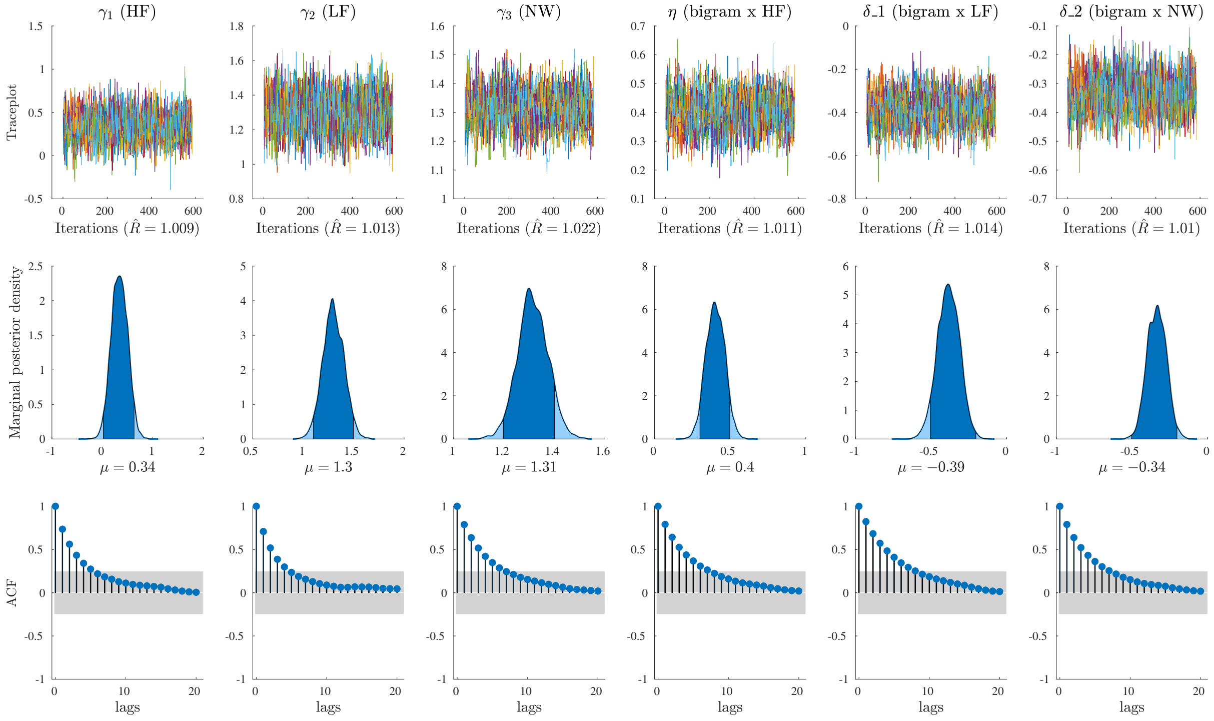

Figure 3: Case study (Model 1): MCMC diagnostics. First row: Traceplot of the chains in different colors and Gelman-Rubin $\hat{R}$ index. Second row: Marginal posterior densities with posterior means μ and 95% HDPI. Third row: Autocorrelation function (ACFs) for the collapsed chains.

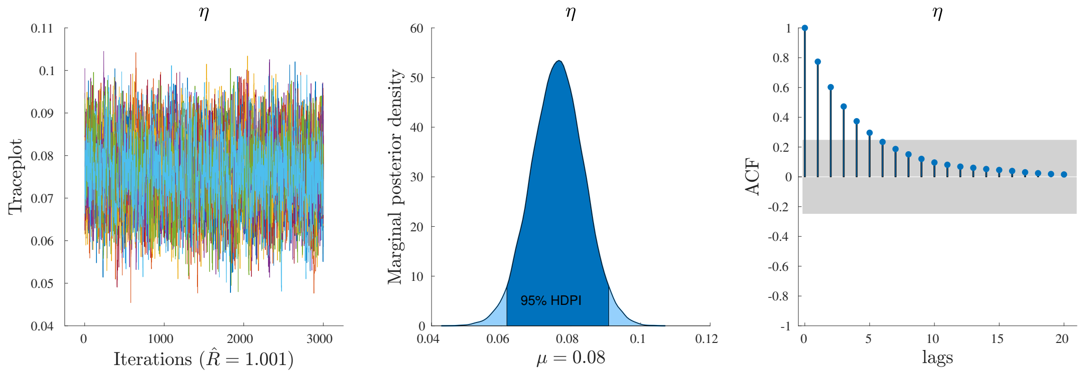

Figure 4: Case study (Model 3): MCMC diagnostics. First row: Traceplot of the chains in different colors and Gelman-Rubin $\hat{R}$ index. Second row: Marginal posterior densities with posterior means μ and 95% HDPI. Third row: Autocorrelation function (ACFs) for the collapsed chains.

2.2 Fit of the model

The adequacy of the state-space model to reproduce the observed data was computed by means of a simulation-based approach. In particular, given the posteriors of the parameters $\hat{\boldsymbol{\beta}}$ and filtered/smoothed latent states $\hat{\mathbf{Z}}$, M new (simulated) datasets $(\mathbf{Y}_1^*, \ldots, \mathbf{Y}_M^*)$ were generated according to the estimated model structure. For each new dataset, the AoR discrepancy measure was computed:

$$\text{AoR} = 100 \cdot \frac{\min\left(||\mathbf{y}^\dagger||, ||\text{vec}(\mathbf{Y})||\right)}{\max\left(||\mathbf{y}^\dagger||, ||\text{vec}(\mathbf{Y})||\right)} \quad \text{with} \quad \mathbf{y}^\dagger = \frac{\text{vec}(\mathbf{Y}_m^*)^T \text{vec}(\mathbf{Y})}{||\text{vec}(\mathbf{Y})||} \cdot \frac{\text{vec}(\mathbf{Y})}{||\text{vec}(\mathbf{Y})||} \qquad m = 1, \ldots, M$$

In addition, to further evaluate the amount of reconstruction, we computed two kind of AoR measures:

- **Overall AoR:** percentage of data reconstruction based on the overall $I \times J \times N$ observed matrix $\mathbf{Y}$.

- **By-subject AoR:** percentage of data reconstruction based on the $J \times N$ observed matrix $\mathbf{Y}_i$ for each subject $i = 1, \ldots, I$. The index allows for evaluating the adequacy of the model to reconstruct the individual-based set of trajectories.

The simulation was performed on a (remote) HPC machine based on 16 cpu Intel Xeon CPU E5-2630L v3 1.80GHz, 16x4GB Ram. Figures 5-7 show the results of AoR indices for the three models. Figure 8 shows the observed mean trajectories for each individual $\mathbf{Y}_i$, $i = 1, \ldots, I$ against the predicted trajectories by the three models.

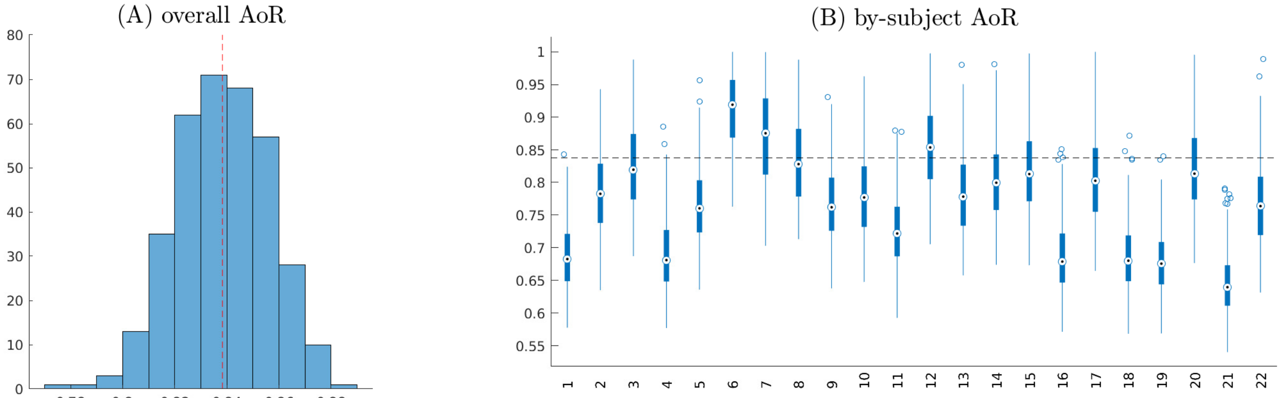

Figure 5: Goodness of fit (Model 1): (A) Overall amount of reconstruction and (B) By-subject amount of reconstruction. Note that dotted lines represent the mean of the overall AoR index.

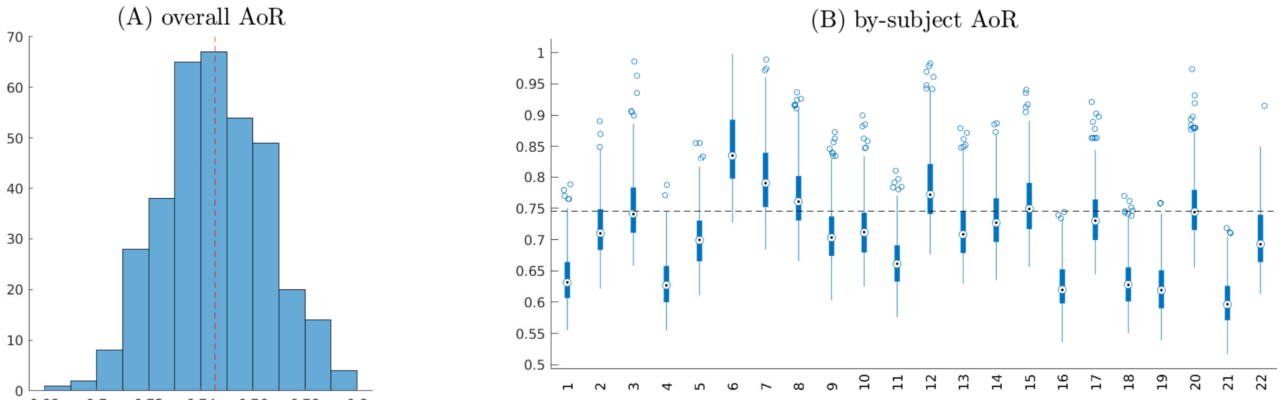

Figure 6: Goodness of fit (Model 2): (A) Overall amount of reconstruction and (B) By-subject amount of reconstruction. Note that dotted lines represent the mean of the overall AoR index.

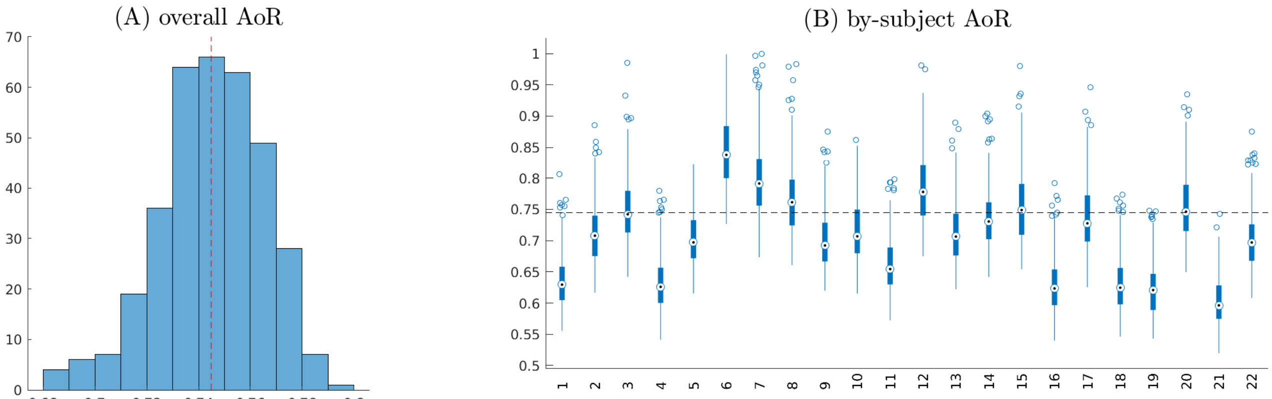

Figure 7: Goodness of fit (Model 3): (A) Overall amount of reconstruction and (B) By-subject amount of reconstruction. Note that dotted lines represent the mean of the overall AoR index.

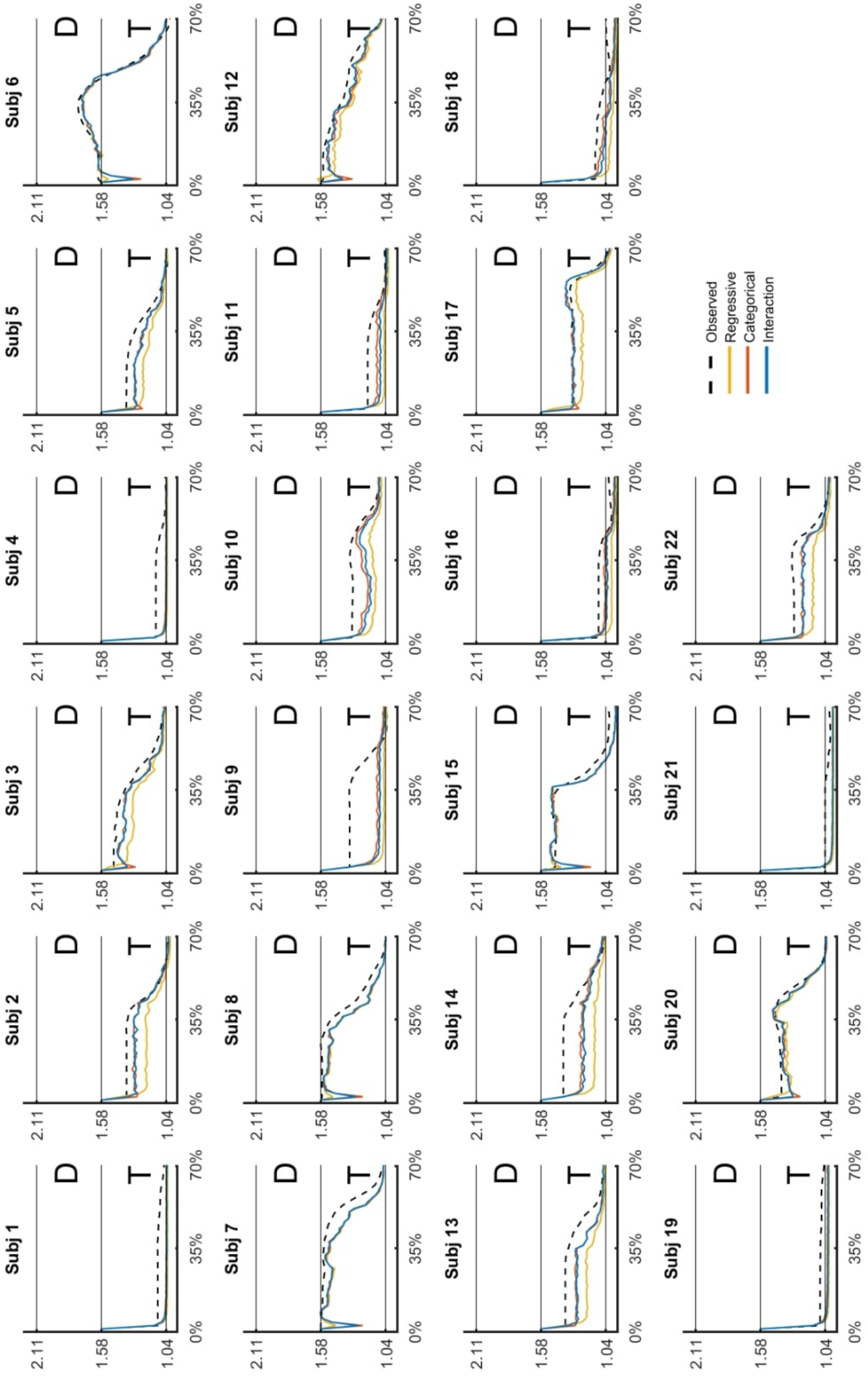

Figure 8: Goodness of fit: Observed and predicted individual (mean) trajectories for each of three models. Note that dotted lines represent observed trajectories whereas the upper and lower halves indicate distractor (D) and target (T) hemispaces, respectively.
